# Pulmonary arteriovenous malformations in patients with previous brain abscess: a cross‐sectional population‐based study

**DOI:** 10.1111/ene.16176

**Published:** 2023-12-08

**Authors:** Jacob Bodilsen, Trine Madsen, Christian Thomas Brandt, Katrine Müllertz, Lothar Wiese, Semra Turan Demirci, Hannah Elena Suhrs, Lykke Larsen, Sabine Ute Alice Gill, Birgitte Rønde Hansen, Brian Nilsson, Lars Haukali Omland, Emil Fosbøl, Anette Drøhse Kjeldsen, Henrik Nielsen, DASGIB Study Group

**Affiliations:** ^1^ Department of Infectious Diseases Aalborg University Hospital Aalborg Denmark; ^2^ Department of Cardiology Aalborg University Hospital Aalborg Denmark; ^3^ Department of Infectious Diseases Zealand University Hospital Roskilde Denmark; ^4^ Department of Clinical Medicine Copenhagen University Copenhagen Denmark; ^5^ Department of Cardiology Nordsjællands Hospital Hillerød Hillerød Denmark; ^6^ Department of Cardiology Zealand University Hospital Roskilde Denmark; ^7^ Department of Infectious Diseases Odense University Hospital Odense Denmark; ^8^ Department of Cardiology Odense University Hospital Odense Denmark; ^9^ Department of Infectious Diseases Hvidovre University Hospital Hvidovre Denmark; ^10^ Department of Cardiology Hvidovre University Hospital Hvidovre Denmark; ^11^ Department of Infectious Diseases Copenhagen University Hospital, Rigshospitalet Copenhagen Denmark; ^12^ Department of Cardiology, Heart Centre University Hospital of Copenhagen, Rigshospitalet Copenhagen Denmark; ^13^ Institute for Clinical Medicine University of Copenhagen Copenhagen Denmark; ^14^ Department of Otorhinolaryngology Odense University Hospital Odense Denmark; ^15^ Institute for Clinical Medicine Aalborg University Aalborg Denmark

**Keywords:** brain abscess, cerebral abscess, hereditary haemorrhagic telangiectasia, HHT, PAVM, PFO, pulmonary arteriovenous malformation, shunt

## Abstract

**Background and purpose:**

Pulmonary arteriovenous malformations (PAVMs) may cause recurrent brain abscess. The primary aim was to determine the prevalence of PAVM amongst survivors of brain abscess. The proportion with cardiac right‐to‐left shunts was also assessed post hoc.

**Methods:**

This was a cross‐sectional population‐based study of adult (≥18 years) survivors of cryptogenic bacterial brain abscess in Denmark from 2007 through 2016. Patients were invited for bubble‐echocardiography to detect vascular right‐to‐left shunting and, if abnormal, subsequent computed tomography thorax for diagnosis of PAVM. Data are presented as *n*/*N* (%) or median with interquartile range (IQR).

**Results:**

Study participation was accepted by 47/157 (30%) eligible patients amongst whom two did not appear for scheduled bubble‐echocardiography. The median age of participants was 54 years (IQR 45–62) and 19/57 (33%) were females compared with 59 years (IQR 48–68, *p* = 0.05) and 41/85 females (48%, *p* = 0.22) in non‐participants. Bubble‐echocardiography was suggestive of shunt in 10/45 (22%) participants and PAVM was subsequently confirmed by computed tomography in one patient with grade 1 shunting. The corresponding prevalence of PAVM was 2% (95% confidence interval 0.06–11.8) amongst all examined participants. Another 9/45 (20%) were diagnosed with patent in persistent foramen ovale (*n* = 8) or atrial septum defect (*n* = 1), which is comparable with the overall prevalence of 25% amongst adults in the Danish background population.

**Conclusions:**

Undiagnosed PAVM amongst adult survivors of cryptogenic bacterial brain abscess is rare but may be considered in select patients. The prevalence of cardiac right‐to‐left shunts amongst brain abscess patients corresponds to the prevalence in the general population.

## INTRODUCTION

Brain abscess remains a serious infection with a case fatality rate of 8%–25% and neurological sequelae in 20%–70% of survivors [1, 2]. The aetiology is primarily oral cavity bacteria (frequently polymicrobial) followed by *Staphylococcus aureus* [2]. Risk factors for brain abscess include recent neurosurgery, contiguous or dental infections, immunosuppression and head trauma [3]. However, approximately 20%–30% of patients have cryptogenic abscesses, that is, no identified predisposing factor [1, 2].

Previous studies suggest that patients with cryptogenic brain abscesses caused by oral cavity bacteria may have undiagnosed pulmonary arteriovenous malformations (PAVMs), usually as part of the hereditary haemorrhagic telangiectasia syndrome (HHT) [4, 5]. This may lead to right‐to‐left vascular shunting of transient bacteraemia and development of brain abscess. A cross‐sectional study used a registry of 445 patients with HHT in the UK from 2005 to 2016 and found 37 patients (8.3%) with brain abscess either before (29/37, 78%) or after diagnosis of PAVM [4]. Corresponding estimates were 7.8% amongst HHT patients with PAVM in Denmark [6]. The association between HHT and brain abscess has also been confirmed in other retrospective cohort studies [5, 7, 8, 9, 10].

However, data on systematic screening of patients with brain abscess for PAVM or HHT are lacking. Thus, the aim was to ascertain the prevalence of undiagnosed HHT in patients with previous brain abscess in Denmark from 2007 through 2016 by inviting survivors to a diagnostic work‐up for PAVMs. In post hoc analysis, the prevalence of cardiac right‐to‐left shunts was also accounted for.

## METHODS

### Setting and study population

This population‐based cross‐sectional study identified all adults (≥18 years of age) diagnosed with brain abscess in Denmark from 2007 through 2016 in the Danish National Patient Registry as described previously (Supplementary Material) [2]. The registry holds administrative information on all hospital contacts in Denmark including diagnosis codes according to the International Classification of Diseases version 10. The study included all patients from North Denmark Region, South Denmark Region, Region Zealand and the Capital Region of Denmark comprising approximately 80% of the entire Danish population.

Next, each patient was assessed for eligibility by manual review of the medical records. Exclusion criteria comprised previous diagnosis of PAVM, other known predisposing conditions or brain abscesses caused by parasites, tuberculosis, nocardiosis or fungi. Patients were invited to participate in the study by letter with a reminder sent after a couple of months if they had not responded. Finally, non‐respondents were contacted by telephone. Survivors of brain abscess were required to be able to give informed consent to participate in the study.

### Diagnostic work‐up for PAVM and HHT

First, participants were examined by bubble‐echocardiography (i.e., injection of agitated saline containing microscopic air bubbles) to detect cardiovascular right‐to‐left shunting such as patent foramen ovale (PFO) or PAVM. Bubble‐echocardiography has a high sensitivity for detection of PAVM and is recommended as a validated screening procedure for PAVM amongst HHT patients [11, 12]. Abnormal results were categorized as grade 1 (<10 bubbles in the left ventricle during the first heartbeat after injection of agitated saline), grade 2 (moderate amounts of bubbles), grade 3 (large amounts of bubbles, but less than on the right side) and grade 4 (similar opacification of the right and left ventricle). Next, participants with suspected PAVM were referred for computed tomography of the chest (5 mm slices) for further clarification.

### Patient data

Information on clinical characteristics during admission for brain abscess was obtained from the medical records. Oral cavity bacteria were defined as non‐haemolytic streptococci, Fusobacterium, Actinomyces, Haemophilus and similar species.

### Statistical analysis

Categorical variables were presented as *n*/*N* (%) with 95% confidence interval (CI) for the primary outcome, that is, the prevalence of PAVM. Continuous variables were presented as median with interquartile range (IQR). Stata MP version 17 (StataCorp) was used for statistical analyses.

### Ethical considerations

The study was approved to be carried out in adults by the Ethics Committee (N‐20170067) and the Danish Data Protection Agency (2008‐58‐0028).

## RESULTS

The entire cohort comprised 315 cases of brain abscess in 313 individuals including two patients (0.6%) already diagnosed with HHT and one patient with nasopharyngeal cancer who had been hospitalized with three separate episodes of brain abscess. Of these, 157 adult survivors with a potential unclear source of bacterial brain abscess were eligible for study inclusion, which was accepted by 47/157 (30%) patients (Table 1).

**TABLE 1:** Baseline characteristics of survivors of cryptogenic bacterial brain abscess according to response to invitation for screening for pulmonary arteriovenous malformations.

	Participants, *N* = 47	Non‐participants, *N* = 110	*p* value*
	*n*/*N* (%) or median (IQR)	*n*/*N* (%) or median (IQR)	
Age, years	55 (45–62)	59 (49–69)	0.05
Females	17 (36)	53 (48)	0.22
No physical impairment before brain abscess	39 (83)	79/107 (74)	0.30
Full‐time work or study before brain abscess	33/45 (73)	40/101 (40)	<0.001
Immuno‐compromised^a^	10 (21)	29 (26)	0.64
Alcohol abuse	3 (6)	11 (10)	0.56
Neurological deficit at admission	36 (77)	97 (88)	0.11
Cranial nerve deficit	9/40 (23)	30/92 (33)	0.33
Extremity paresis or sensory deficit	10/38 (26)	53/97 (55)	0.006
Aphasia	7/38 (18)	28/95 (29)	0.28
Brain abscess^b^			
Multiple	10/45 (22)	24/109 (22)	0.86
Size, cm	2.6 (1.9–3.1)	3.0 (2.0–3.5)	0.23
Frontal	14 (30)	48 (44)	0.15
Temporal	12 (26)	22 (20)	0.58
Parietal	16 (34)	36 (33)	1.0
Occipital	14 (30)	21 (19)	0.21
Cerebellum and other brain structures	6 (13)	23 (21)	0.33
Pathogen			
Oral cavity bacteria	38 (81)	85 (77)	0.78
Unknown	7 (14)	9 (8)	0.25
Aspiration or excision of brain abscess	40 (85)	93 (85)	0.89
Intensive care unit	14 (30)	22/109 (20)	0.26
Minor or no sequelae at discharge	19 (40)	35 (32)	0.39

*Note*: Categorical variables were compared by Fisher's exact test or chi‐squared (Yates) as appropriate. Continuous variables were compared using the Mann–Whitney rank sum test.

Abbreviation: IQR, interquartile range.

^a^ Immuno‐compromised was defined as alcohol or intravenous substance abuse, solid organ transplant recipients, solid or haematological cancer, diabetes mellitus, asplenia, human immunodeficiency virus or primary immune deficiency.

^b^ Several areas of the brain may be involved in patients with multiple abscesses.

* *p* value <0.05 was considered statistically significant.

The participants had a median age of 55 years (IQR 45–62) and 17/47 (33%) were females compared with 59 years (IQR 49–69, *p* = 0.05) and 53/110 females (48%, *p* = 0.22) in non‐participants. Prior to admission for brain abscess, 33/45 (73%) participants were working full‐time versus 40/101 (40%, *p* ≤ 0.001) non‐participants. Extremity paresis or sensory nerve deficit at admission for brain abscess was reported in 10/38 (26%) participants and 53/97 (55%, *p* = 0.006) non‐participants. Otherwise, characteristics of brain abscesses (number, size and neuroanatomic distribution), pathogens, neurosurgical treatment and outcome were comparable between the two groups.

Bubble‐echocardiography was carried out in 45/47 (96%) participants (two did not appear for scheduled examination) and was suggestive of shunt in 10/45 (22%) (Table 2). Another 9/45 (20%) were diagnosed with PFO (*n* = 8) or atrial septum defect (*n* = 1).

**TABLE 2:** Results of screening for pulmonary arteriovenous malformations amongst 45 survivors of cryptogenic bacterial brain abscess.

	Participants, *N* = 45^a^	Percentage
Bubble‐echocardiography		
Normal	26	58
Suggestive of PAVM^a^	10	22
Grade 1 (i.e., <10 bubbles)	6	60
Grade 2 (i.e., moderate amounts of bubbles)	3	30
Grade 3 (i.e., large amounts of bubbles)	1	10
Patent foramen ovale or atrial septum defect	9	20
CT chest	14	28
Normal	13	93
PAVM	1	7

Abbreviations: CT, computed tomography; PAVM, pulmonary arteriovenous malformation.

^a^ 2/47 (4%) did not appear for scheduled bubble‐echocardiography.

All 10 participants with bubble‐echocardiography indicating PAVM as well as another four with PFO were referred for computed tomography of the chest (*n* = 14) and PAVM was diagnosed in 1/45 participants yielding a prevalence of 2% (95% CI 0.06–11.8). The participant had a parieto‐occipital brain abscess due to oral cavity bacteria and bubble‐echocardiography showed grade 1 shunting.

## DISCUSSION

This cross‐sectional population‐based study aimed to determine the prevalence of undiagnosed PAVM in adult survivors with an unclear source of bacterial brain abscess. Study participation was low (30%) and PAVM was only found in one participant yielding a prevalence of 2% (95% CI 0.06–11.8) amongst the examined individuals.

The observed prevalence of PAVM amongst patients with brain abscess in the current study is in line with previous reports [5, 8, 13] but lower than some studies of selected study populations or with unclear denominators [7, 9, 10]. Other reasons for the discrepancies may include variations in the prevalence of PAVM/HHT between countries. A French multi‐centre study found that 21/26 (75%) patients with brain abscess and PAVM were subsequently diagnosed with HHT at a mean of 81 months (SD 64) after treatment for brain abscess [7]. Similarly, a Danish registry‐based study of 1384 patients found that only 2/17 (12%) with HHT were known with this condition before hospitalization for brain abscess [13]. Interestingly, patients with brain abscess and underlying PAVM frequently present with single, supratentorial brain abscess caused by oral cavity bacteria. Thus, HHT should be considered in such patients with unclear reason(s) for development of brain abscess, especially in recurrent brain abscess and/or if combined with a history of epistaxis, gastro‐intestinal haemorrhage or arteriovenous malformations elsewhere in the body. PFO has also been suggested as a risk factor for brain abscess by right‐to‐left vascular shunting of transient bacteraemia [14], but the occurrence in the current study was comparable to prior estimates of ≈25% in adults and does not provide convincing support for this notion [15].

Limitations of the current study include a low response rate amongst eligible patients. Furthermore, some patients with undiagnosed PAVM or HHT may have died between the time of discharge for brain abscess and study onset. Misclassification of patients excluded due to, for example, ear−nose−throat infections or other predisposing conditions cannot be ruled out and some patients may have several risk factors for brain abscess. Common for all these limitations is a potential underestimation of PAVM as a risk factor for brain abscess.

In conclusion, undiagnosed PAVM amongst adult survivors of cryptogenic bacterial brain abscess is rare but may be considered in selected patients.

## CONFLICT OF INTEREST STATEMENT

All authors declare no conflicts of interests.

## Data Availability

The data that support the findings of this study are available on request from the corresponding author. The data are not publicly available due to privacy or ethical restrictions.
